# Precision Medicine in Multiple Sclerosis: Future of PET Imaging of Inflammation and Reactive Astrocytes

**DOI:** 10.3389/fnmol.2016.00085

**Published:** 2016-09-15

**Authors:** Pekka Poutiainen, Merja Jaronen, Francisco J. Quintana, Anna-Liisa Brownell

**Affiliations:** ^1^Athinoula A Martinos Biomedical Imaging Center, Department of Radiology, Massachusetts General Hospital, Harvard Medical SchoolCharlestown, MA, USA; ^2^Ann Romney Center for Neurologic Diseases, Brigham and Women's Hospital, Harvard Medical SchoolBoston, MA, USA

**Keywords:** multiple sclerosis, inflammation, neuroreceptors, positron emission tomography, precision medicine, microglia, astrocyte

## Abstract

Non-invasive molecular imaging techniques can enhance diagnosis to achieve successful treatment, as well as reveal underlying pathogenic mechanisms in disorders such as multiple sclerosis (MS). The cooperation of advanced multimodal imaging techniques and increased knowledge of the MS disease mechanism allows both monitoring of neuronal network and therapeutic outcome as well as the tools to discover novel therapeutic targets. Diverse imaging modalities provide reliable diagnostic and prognostic platforms to better achieve precision medicine. Traditionally, magnetic resonance imaging (MRI) has been considered the golden standard in MS research and diagnosis. However, positron emission tomography (PET) imaging can provide functional information of molecular biology in detail even prior to anatomic changes, allowing close follow up of disease progression and treatment response. The recent findings support three major neuroinflammation components in MS: astrogliosis, cytokine elevation, and significant changes in specific proteins, which offer a great variety of specific targets for imaging purposes. Regardless of the fact that imaging of astrocyte function is still a young field and in need for development of suitable imaging ligands, recent studies have shown that inflammation and astrocyte activation are related to progression of MS. MS is a complex disease, which requires understanding of disease mechanisms for successful treatment. PET is a precise non-invasive imaging method for biochemical functions and has potential to enhance early and accurate diagnosis for precision therapy of MS. In this review we focus on modulation of different receptor systems and inflammatory aspect of MS, especially on activation of glial cells, and summarize the recent findings of PET imaging in MS and present the most potent targets for new biomarkers with the main focus on experimental MS research.

## Introduction

Multiple sclerosis (MS) is the most common disabling neurologic disease of young people, afflicting approximately a quarter of million Americans (Anderson et al., 1992; Islam et al., 2006; Brody, 2012; Ransohoff et al., 2015). It occurs more in women than in men by a ratio of nearly 2 to 1, and it strikes most often between the ages of 20 and 40 (Compston and Coles, 2008). MS results from the immune-driven demyelination of the central nervous system (CNS), which leads to axonal damage and progressive loss of neurological functions (Sofroniew and Vinters, 2010; Malpass, 2012; Sofroniew, 2015). Based on clinical characteristics, MS pathology can be divided into three different disease courses: relapsing-remitting (RR), secondary progressive (SP), and primary progressive (PP) (Goodin, 2014). Initially, most MS patients exhibit a RR-MS disease course (Morales et al., 2006), experiencing heterogeneous symptoms such as ataxia, visual disturbances, paresthesia, and muscle weakness (Ellwardt and Zipp, 2014). However, eventually the majority of these patients develop SP-MS characterized by the progressive and irreversible accumulation of neurological disability (Lublin and Reingold, 1996). PP-MS patients have continuous disease progression from onset, without relapses or remissions (Morales et al., 2006; Lopez-Diego and Weiner, 2008).

**Figure 1:**
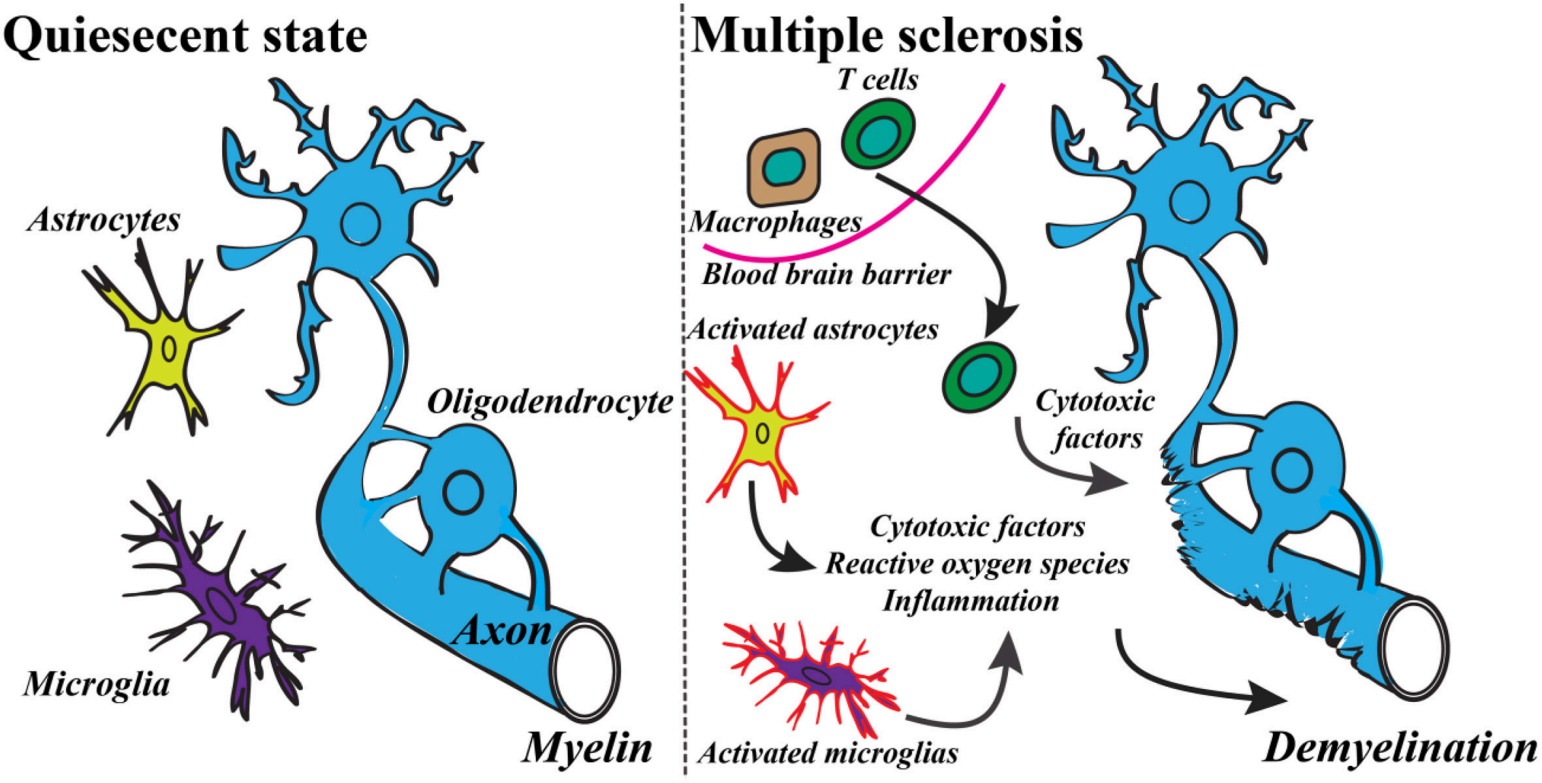
**Basic mechanism of the development of MS includes a variety of inflammatory responses and activation of specific cell types**. Modified from Criste et al. (2014) and Friese et al. (2014).

Recent findings of the innate and the adaptive immune system of CNS have shaken up the classical view of MS as being strictly an autoimmune disease of the white matter (Weiner, 2008; Gandhi et al., 2010; Hemmer et al., 2015). The studies have revealed the important role of infiltrating immune cells from the periphery as well as the role of resident activated glial cells leading ultimately to the T cells and macrophages reaction against myelin (see Figure 1) (Frohman et al., 2006; Compston and Coles, 2008). These advances have switched the focus of MS research toward neurodegenerative aspects of the disease, occurring early in the pathological process (Kiferle et al., 2011). Despite the recent progresses in the field of MS therapeutic strategies there is no curative treatment for progressive MS (Lopez-Diego and Weiner, 2008; Derwenskus and Lublin, 2014). Therefore, identifying new specific biomarkers for MS could reveal new potential drug targets and diagnostic markers. Moreover, there is an unmet clinical need for methods to monitor different mechanisms of disease pathogenesis in MS patients, therefore advanced non-invasive molecular imaging technologies are needed to expand our understanding of the controversial aspects of the MS pathology (Kiferle et al., 2011; Jacobs and Tavitian, 2012).

Historically, MRI has overruled other imaging technologies in the diagnosis of MS (Traboulsee and Li, 2006; Barkhof and Filippi, 2009). The classical McDonald criterion for MS diagnosis requires objective dissemination of lesions in time and space (Filippi and Rocca, 2011). The literature analysis has shown that the sensitivity of MRI has been between 35 and 100%, and specificity has been between 36 and 92% depending on the research protocol (Schäffler et al., 2011; Tillema and Pirko, 2013). Overall, T2-weighted MRI is effective way to detect MS lesions, but because the signal reflects the water content, it does not provide reliable information about the myelin content (Ge, 2006; Poloni et al., 2011). T1-weighted imaging together with contrast agents such as gadolinium-DTPA has increased the lesion detection sensitivity, however, signal frequency is associated with the opening of the blood brain barrier (BBB; Lund et al., 2013). This leads to the problem that MRI can vary greatly in terms of sensitivity and specificity, especially in MS-related pathological pathways (Barkhof et al., 2009; Lövblad et al., 2010). Early diagnosis and treatment is effective for the therapy and decreases the financial burden of the disease (Noyes et al., 2011; Guo et al., 2014). The annual mean cost is around $47,000 per MS patient, which arises to a national cost of about $13 billion in US per year (Olek, 2012). The disease modifying treatments (DMTs, typically used first-line interferon betas and glatiramer acetate) have been available for the last 25 years and are estimated to account for one third of the total cost. Unfortunately, these treatments suppress the disease only for a few years (Hartung et al., 2015) and the spectrum of treatment options is narrow (Oh and O'Connor, 2015). Conventional MRI gives anatomical information from the progressed lesions in the brain of MS patients but lacks the power to provide target for drug discovery and more specific molecular markers when compared to imaging modalities like positron emission tomography (PET; Filippi et al., 2012; Matthews et al., 2012).

PET research field is emerging and the researchers have been successful in developing novel tracers for multiple different aspects of MS to enhance understanding the pathophysiology of the disease. In this review, we summarize PET imaging in MS research and introduce some of the most potent imaging targets and applications that have been successfully investigated in inflammation and which can be implemented especially to astrocyte activation related pathways, which are presently of high interest in MS research (Maragakis and Rothstein, 2006; Nair et al., 2008; Miljković et al., 2011; Nash et al., 2011; Mayo et al., 2014).

## PET imaging techniques

**Figure 2:**
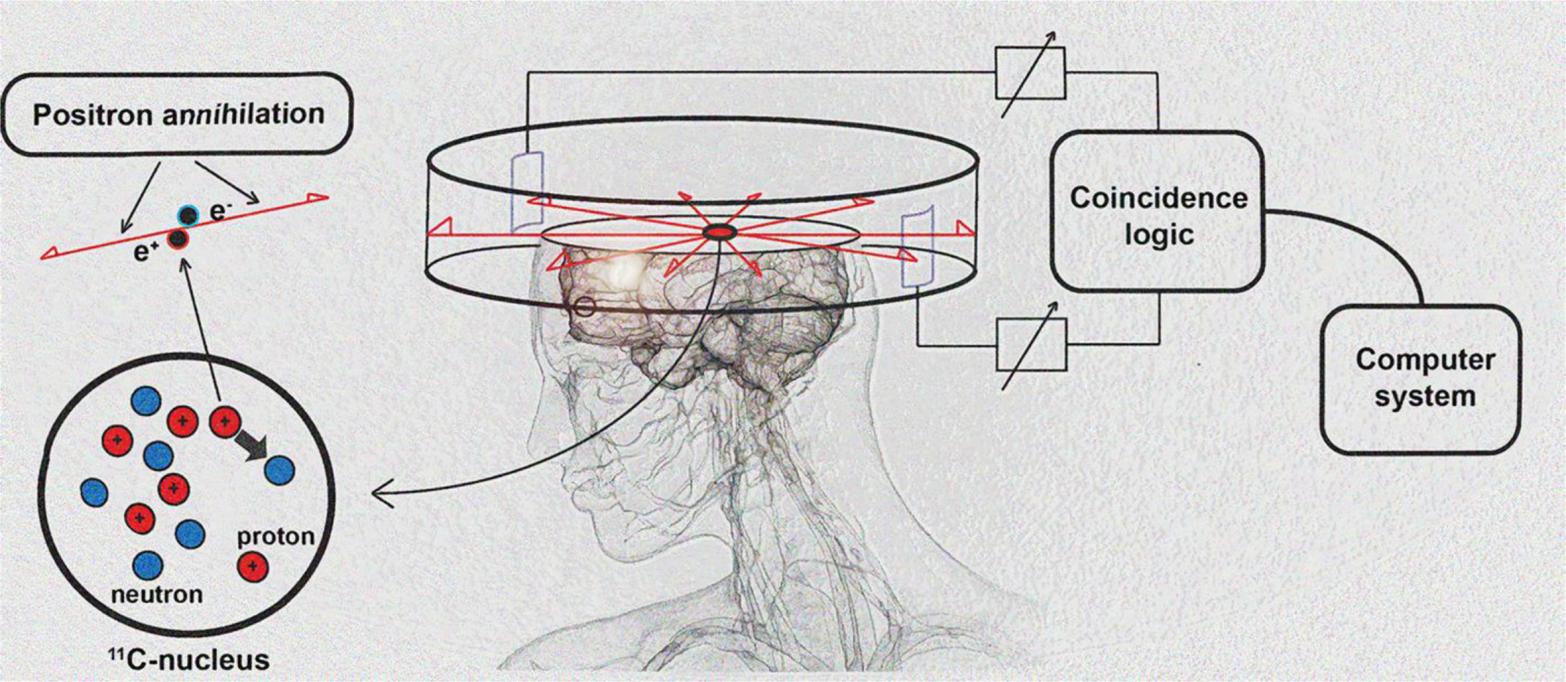
**Schematic diagram of positron detection**. Modified from Brownell (2008).

PET imaging is based on detection of isotope labeled tracers, which emit beta radiation (see Table 1). These tracers are administered into the subjects to monitor underlying biological processes (Kiferle et al., 2011). The radioisotope undergoes positron emission decay and emits positron, which travels into surrounding tissue until it interacts with an electron and the annihilation process takes part (see Figure 2). The formed two photons travel in approximately opposite directions and can be detected with the imaging device as a coincidence pair. Each detected coincident forms a line of response (LOR) where the point of origin is the location of annihilation event. The combination of LORs can be used for reconstruction of images to provide 3-dimensional (3D) distribution of the radiolabeled tracer (Gambhir, 2002).

**Table 1:** **Properties of discussed positron emitting radio-isotopes**.

**Isotope**	**Half-life (min)**	**Production method**	**Positron range in (mm)**	**Maximum positron energy (MeV)**
11_C_	20.3	Cyclotron	1.1	0.96
13_N_	9.97	Cyclotron	1.5	1.19
18_F_	109.8	Cyclotron	0.6	0.64
64_Cu_	764	Cyclotron	0.6	0.65
68_Ga_	67.8	Generator	2.9	1.89
82_Rb_	1.26	Generator	5.9	3.15

*Saha et al., 1994; Partridge et al., 2006; Miller et al., 2008; Jødal et al., 2012*.

The clinical history of positron emission techniques started in 1952 when Gordon Brownell was able to localize brain tumors from patients (Brownell and Sweet, 1953). Further technical progression led to 3D tomographical positron imaging by 1971 (Pizer et al., 1971). However, even today PET suffers from high cost because the production of radiopharmaceutical agents increase the imaging cost compared to CT or MRI, both which became available later. After the development of [^18^F]fluorodeoxy glucose ([^18^F]FDG) PET imaging received more significant clinical role especially in oncological diagnosis (Portnow et al., 2013). In MS however, MRI has been regarded as the golden standard in assessing patients (Filippi et al., 2012; Miller et al., 2012). Combined PET/MRI imaging with high specificity to MS lesions, would have a potential to become a practical tool in clinics to follow up the treatment of MS patients and increase cost-effectiveness. This approach could reveal an optimized treatment regimen; increase the treatment effectiveness and safety of patients, especially in early stage and patients with aggressive disease (Catana et al., 2013). When comparing these two imaging techniques, PET imaging has at least four major advantages over conventional MR imaging: (Massoud, 2003; Kiferle et al., 2011; Poloni et al., 2011; Jacobs and Tavitian, 2012; Miller et al., 2012; Torigian et al., 2013; Faria Dde et al., 2014; Jadvar and Colletti, 2014; Bodini et al., 2015) (1) Specific information of disease mechanism and molecular contributors, (2) Enhance development of new medicines and therapeutic targets, (3) Efficient allocation of new costly therapeutics and personalized medicine, and (4) Improved prognostic method for the MS patients.

Although the first clinical positron emission imaging studies were done over 60 years ago, the spectrum of applications of PET imaging is still limited due to the high cost and lack of validated traces and state-of-the-art facilities including availability of cyclotrons and automated radiopharmaceutical production laboratories (Jones et al., 2012). Complete knowledge about pharmacokinetic and pharmacodynamic properties of injected tracers can assure the correct interpretation of the images from preclinical and clinical studies. Overall, PET is an extremely powerful technology and the *in vivo* receptor occupancy can help answer many vital questions in the MS research (Matthews et al., 2012; Bodini et al., 2015). Furthermore, PET offers an opportunity for the detection of enzyme reactions, ligand-receptor interactions, cellular metabolism, cell proliferation, protein-protein interactions, as well as gene and cell therapy (Herschman, 2003; Ono, 2009; Thorek et al., 2013). The development of new PET tracers is challenging because the binding affinity and selectivity of the tracer have to be high and the dissociation must be fast enough to obtain the binding equilibrium in time frame of scan (1–2 h) (Hicks, 2006; Sharma et al., 2010). The tracer should penetrate the BBB, but too lipophilic compound might have strong non-specific binding (Liu et al., 2008). The optimal radiotracer should have minimum amount of unwanted metabolism and fast synthetic method (usually in single half-life of the radioisotope).

**Figure 3:**
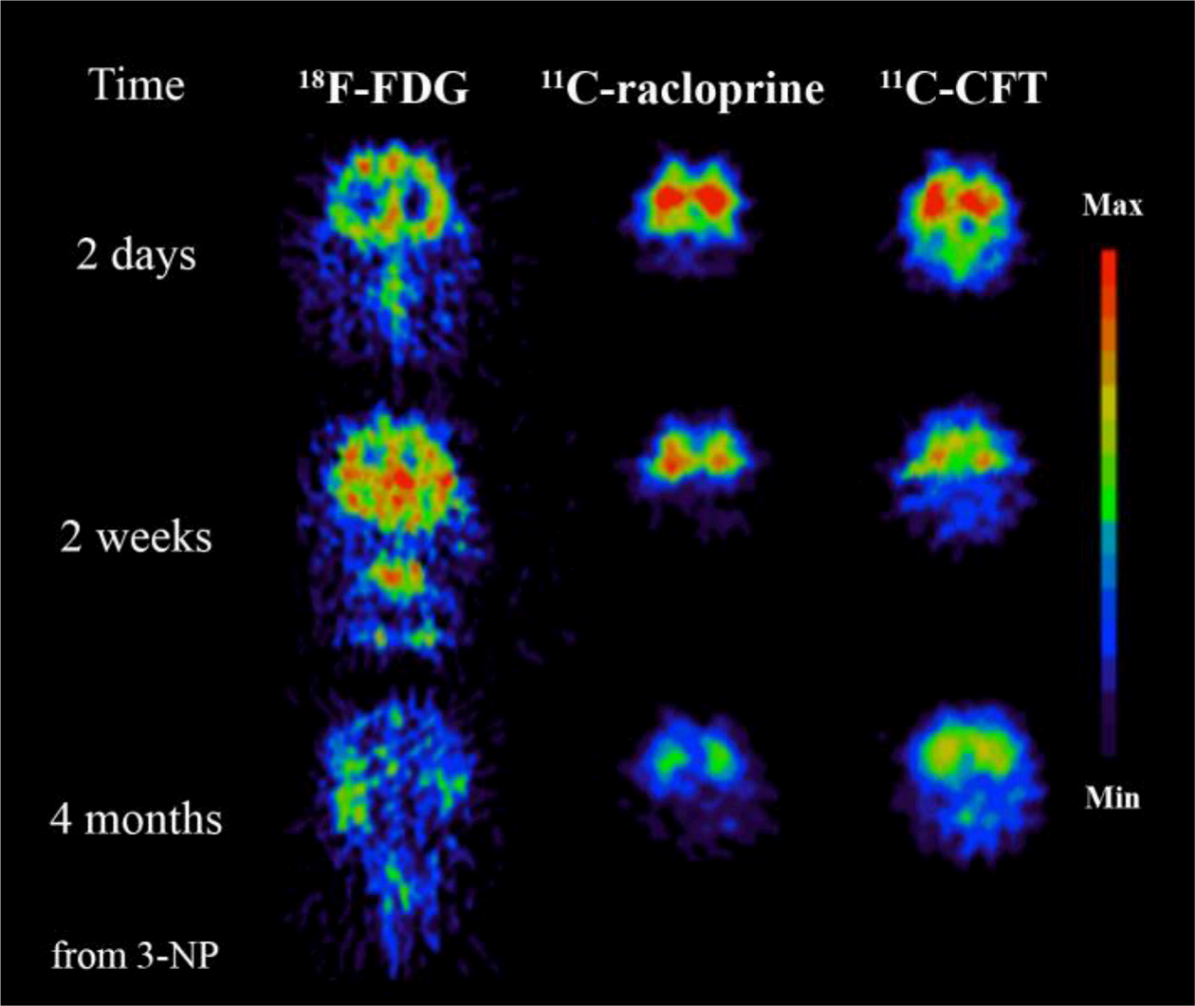
**3-Nitropropionic acid (3-NP, a naturally occurring plant toxin and mycotoxin) could be involved to the development of MS**. This study demonstrates the advantages of PET imaging where specific tracers can be used to reveal different time dependent neurochemical processes. In this case significant decrease of glucose metabolism imaged by 18F-FDG, decrease of dopamine D2 receptor function imaged by 11C-raclopride and decrease of dopamine transporter function imaged with 11C-CFT follow after 3-NP administration. Modified from Brownell et al. (2004).

**Figure 4:**
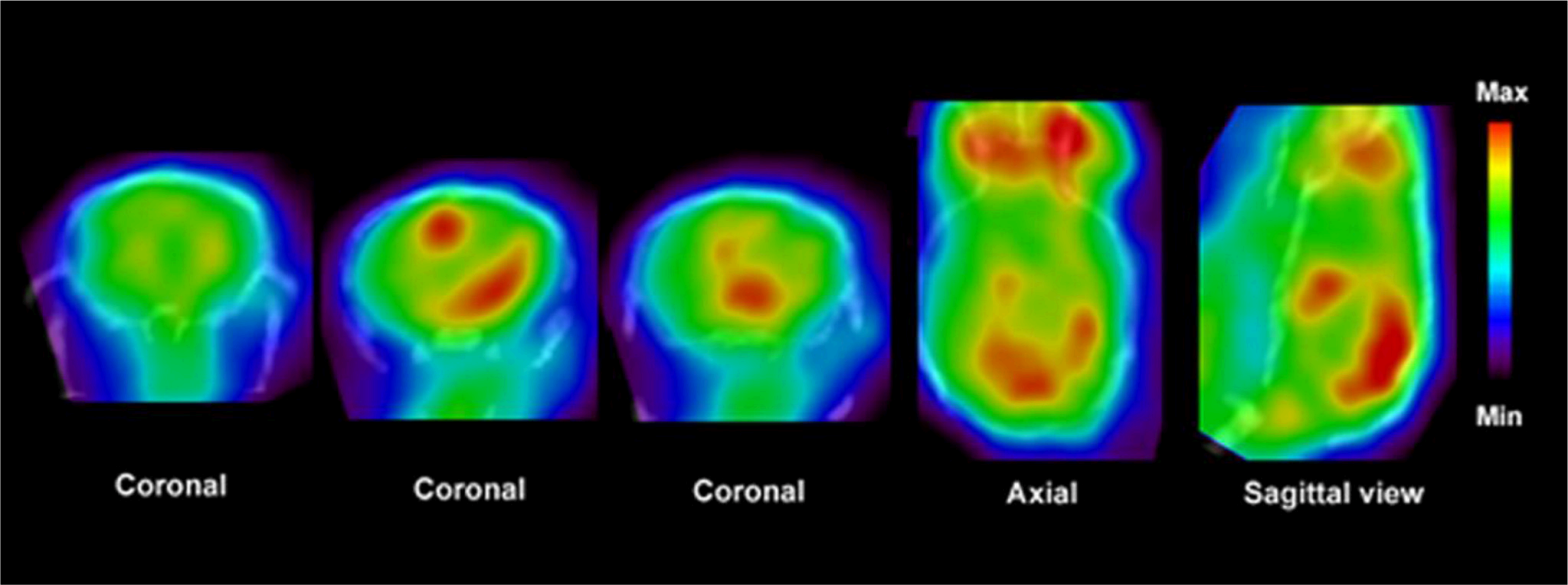
**PET images show distribution of [^**11**^C]PBR28 in the brain of an EAE mouse model**. Fused CT images show the boundaries of the skull. Enhanced accumulation of [^11^C]PBR28 in the hind brain and cerebellum is an indication of regionally activated microglia. Modified from Radu et al. (2007) and Arsenault et al. (2014).

PET imaging systems have been developed also for small animals enhancing significantly basic research. Modern micro-PET instrumentation (resolution < 1 mm) is rapidly expanding the use of non-invasive PET imaging techniques in basic research. These advances have been progressively translated to human studies (Herschman, 2003; Liang et al., 2007; Lancelot and Zimmer, 2010). PET imaging offers tools to evaluate a great variety of molecular aspects of MS and neurodegeneration in animal models as well as in clinics (see Figures 3, 4).

## Preclinical models for MS

Several animal models are used to study mechanisms of disease pathogenesis relevant for MS (Furlan et al., 2009; Denic et al., 2011; Ransohoff, 2012). Experimental autoimmune encephalomyelitis (EAE) is the most vividly used animal model especially to study the inflammation aspects of MS. In this model, rodents are immunized with myelin antigens to activate peripheral antigen specific T-cells, which travel to CNS and induce formation of demyelinating lesion (Baxter, 2007; Constantinescu et al., 2011). Based on the hypothesis that viral infections may cause MS, virus-induced demyelination animal models are also used to study the disease (Gilden, 2005; Owens et al., 2011; Tselis, 2012). A disadvantage of this model is that the experimental disease manifests months after the initial infection (Olson et al., 2001; Fatima et al., 2010). Demyelination and spontaneous remyelination processes relevant to MS are predominantly studied using toxin-induced models (Blakemore and Franklin, 2008). The induction with copper chelating agent, Cuprizone [oxalic acid bis(cyclohexylidene hydrazide)], is one of the frequently used methods, since it is highly reproducible, relatively simple, induces fast demyelination, and the model has spontaneous remyelination after halting the toxin exposure (Torkildsen et al., 2008; Kipp et al., 2009). The focal demyelination lesions are commonly induced with ethidium bromide and lysolecithin (Woodruff and Franklin, 1999). The small size of the disease model increases the technical aspects for imaging technology. In the following chapters we will discuss the current tracers designed to detect the main pathological features of MS.

## PET imaging of axonal degeneration

The complex network of conditions leading to neuroaxonal degeneration and neuronal loss contribute the permanent disability related to MS pathology (Friese et al., 2014). Even though during the earlier days of MS research, axonal loss was considered to be late-occurring, it is now discovered to happen also in the early stages of MS (Trapp and Nave, 2008; Trapp and Stys, 2009). In the earlier phases of the disease axonal damage can occur acutely in the new inflammatory lesions. Whereas later in the disease progression axonal damage is usually related to chronic and demyelinated regions and there is only little if any active inflammation present (Criste et al., 2014). In addition, there is growing amount of evidence from both MRI and histological studies proposing that the axonal degeneration contributes to the development of clinical disability (Edgar and Nave, 2009; Nave, 2010). These interesting facts further highlight the need of specific markers for imaging of disease stage.

Currently, the most promising marker for neuronal integrity is benzodiazepine site on the GABA_A_ receptor (Sigel and Buhr, 1997). Flumazenil, antagonist for benzodiazepine site, has been already labeled with ^18^F and ^11^C (Suzuki et al., 1985; The MICAD Research Team, 2004). Interestingly, in MS patients the axonal reduction has been demonstrated with the use of [^11^C]flumazenil (Barkhof et al., 2009). Furthermore, it has been shown that focal brain inflammation causes reduced GABA_A_ mediated inhibition in neurons (Rossi et al., 2012). In addition, the inhibition is also induced in gray matter in the acute relapsing phases of MS (Rossi et al., 2012). Rossi et al. suggest that neurodegeneration in white and gray matter lesions are accompanied by a loss of GABA_A_ receptors. PET could visualize this with radiolabeled flumazenil. However, this strategy remains yet to be tested.

Another cell type, which suffers from axonal loss during MS, is cholinergic neurons (D'Intino et al., 2005). Degradation of these neurons can, at least party, contribute to the cognitive impairment of the MS patients (Kooi et al., 2011). Interestingly, when assessing the acetylcolinesterase (AChE) activity by [^11^C]MP4A (^11^C-methyl-4-piper-idinylpropionate), an inverse correlation with the activity of AChE and cognitive impairment was observed in MS patients (Virta et al., 2011). This result is contradicting the demonstrated positive response seen in MS patients with AChE inhibitors (Krupp et al., 2004; Tsao and Heilman, 2005). However, it has been hypothesized that the controversial results with increased AChE expression might be due to induction by inflammatory response in glial cells (Virta et al., 2011).

In addition, the reduction of glucose metabolism in the degenerated regions has shown correlation between disease activity, hypo-metabolism and specific cognitive functions during the MS progression (Bakshi et al., 1998). [^18^F]FDG has some valuable characteristics for monitoring cognitive and mental dysfunctions associated with MS (Paulesu et al., 1996; Zarei, 2003; Buck et al., 2012; Colasanti et al., 2014).

## PET imaging of demyelination

Demyelination, the pathological removal of myelin sheaths surrounding the axons, has been thought to be an integral part of axonal degeneration, as chronic CNS demyelination has been demonstrated to lead to axonal pathology and degeneration (Wilkins et al., 2010). However, these two events can happen independently from one another as axonal degeneration has been demonstrated to occur without myelin loss (Nave, 2010) and recently it has been demonstrated that the loss of myelin does not necessarily lead to axonal degeneration (Smith et al., 2013).

Classically in MS, demyelination is thought to cause the axonal dysfunction and disease-related pathogen conditions (Lucchinetti et al., 2000). On the other hand, spontaneous remyelination, executed by oligodendrocytes that mature from oligodendrocyte precursor cells, may occur following demyelination, presumably allowing a partial, if not complete, recovery from disability (Brück, 2005; Compston and Coles, 2008). Both adaptive and innate immune systems control the fine balance between demyelination and remyelination during MS and determine the outcome of the disease (Zhang et al., 2013). However, recently researchers have demonstrated early loss of both neurons and oligodendrocytes, leading to the question whether inflammatory demyelination is primary or secondary in the disease process of MS (Trapp and Nave, 2008). Remyelination is usually seen to occur in the early phases of the disease, whereas in the later phases it fails to recover the demyelinated areas leading to chronic demyelinated lesions (Chang et al., 2002; Kuhlmann et al., 2008).

Several tracers have been developed to target the β-sheet structures of intact myelin (Wu et al., 2006; Mallik et al., 2014). The first tracer, [^11^C]BMB (1,4-bis(p-aminostyryl)-[^11^C]2-methoxy benzene), had significant off-target affinity toward white and gray matter (Stankoff et al., 2006). Some of these downsides have been overcome with Congo red derivatives (e.q. [^11^C]CIC, Case Imaging Compound) and thioflavine-T derivatives (e.q. [^11^C]PIB, N-methyl-[^11^C]2-(4′-methylaminophenyl)-6-hydroxybenzothyazole). Moreover, these tracers have more reliable production and BBB penetration (Wang et al., 2009; Stankoff et al., 2011). Recent comparisons between these series and new [^11^C]MeDAS, (N-[^11^C]methyl-4,4′-diaminostilbene) tracers prefer the latter compound as the most promising ligand so far to detect MS-like lesions and spinal cord imaging (de Paula Faria et al., 2014b). [^11^C]MeDAS has been successfully used to image acute focal neuroinflammation in the brain, lyso-phosphatidyl choline induced focal demyelination in the spinal cord and EAE rodent models of MS (Wu et al., 2013). Furthermore, [^11^C]MeDAS was also able to highlight both demyelination and remyelination processes in cuprizone mouse model (de Paula Faria et al., 2014b). Interestingly, the uptake of [^11^C]MeDAS was not interfered by inflammation (Wu et al., 2013). The current literature suggest that [^11^C]MeDAS is the most preferred PET agent so far to highlight the lesions as well as the myelin content in the spinal cord in motor disability related MS (de Paula Faria et al., 2014a). To this point the only PET tracer used to image myelin in MS patients is [^11^C]PIB, a tracer widely utilized to visualize β-amyloid plaques in Alzheimer's disease (Stankoff et al., 2011; Zhang et al., 2014).

Altogether, PET imaging of myelin integrity shows great potential in animal models of MS. It is interesting to validate these methods in patients, especially now that new remyelination therapies are introduced in clinical trials (Brugarolas and Popko, 2014).

## PET imaging of microglial activation

Neuroinflammation is a common characteristic of numerous neurodegenerative disorders, including MS (Glass et al., 2010). Reactive states of astrocytes (astrogliosis), and microglia (microgliosis), as well as the infiltration of the lymphocytes are the hallmarks of neuroinflammation (Carson et al., 2006). Although factors inducing inflammation vary between CNS related diseases, there is evidence that convergence mechanisms are accountable for the sensing, transduction, and amplification of inflammatory processes that eventually lead to the production of neurotoxic mediators (Glass et al., 2010). In fact, neuroinflammation is a highly dynamic and complex process combining local and systemic reactions of multiple cell types, chemical signals, and signaling pathways to adaptive response for restoring tissue homeostasis (Medzhitov, 2008; Aguzzi et al., 2013; Naegele and Martin, 2014). In the following, we will discuss the PET tracers used to visualize microglial and astrocytic activation.

Microglia are of mesenchymal origin and constantly monitor the extracellular environment as well as interact closely with astrocytes and neurons (Yamasaki et al., 2014; Michell-Robinson et al., 2015). As macrophages in the periphery, microglia are the first line of defense against infections or insults in the CNS (Olson et al., 2001; Hanisch and Kettenmann, 2007; Nau et al., 2014). Upon activation, microglia acquire an amoeboid appearance and secrete pro-inflammatory molecules such as interleukin 1β, interferon γ, and tumor necrosis factor-α (TNFα) (Boche et al., 2013): a classically activated M1 state (Mills et al., 2000; Martinez and Gordon, 2014). The aim of the pro-inflammatory reaction is to clear the hazardous material and correct the inflicted damage (Gordon, 2003; Martinez et al., 2009). Usually, the pro-inflammatory reaction is down-regulated by the anti-inflammatory molecules (Tambuyzer et al., 2009; Scheller et al., 2011). In addition to pro-inflammatory molecules, microglia can release trophic and anti-inflammatory factors such as interleukins 4 and 10 as well as insulin-like growth factor 1 (Cherry et al., 2014). These factors are aimed to contribute to the repair and limitation of the inflammation (Mantovani et al., 2004; Hanisch and Kettenmann, 2007; Michelucci et al., 2009). Astrocytes and inflammatory T-cell subsets surrounding microglia influence the state of microglia, and determine whether they are releasing pro- or anti-inflammatory factors (Shih, 2006; Goverman, 2009; Mayo et al., 2012, 2014; Quintana et al., 2014).

The role of microglial activation in MS progression has remained enigmatic (Correale, 2014). However, several theories have been offered. The first theory suggests that inflammatory processes similar to those observed in RR-MS cause the brain damage (Kutzelnigg and Lassmann, 2014). However, during the progressive disease stages, a microenvironment is created within the brain favoring the homing and retention of inflammatory cells, finally resulting in the failure of disease-modifying therapies (Frischer et al., 2009). According to the second theory, MS starts out as an inflammatory disease and after several years, neurodegeneration, a process autonomous of inflammatory response, becomes the mechanism responsible for progression of the disease (Meuth et al., 2008; Kutzelnigg and Lassmann, 2014). Finally, MS could be seen primarily as a neurodegenerative disease, where inflammation occurs as a secondary response, augmenting and modifying progressive stages (Kassmann et al., 2007; Fitzner and Simons, 2010). Needless to say, these theories are not mutually exclusive. Furthermore, it has been postulated that the lack of understanding the exact microglial function during course of MS, has led to the absence of therapies for SP-MS (Correale, 2014). Altogether, this clearly demonstrates the need for a consensus and better understanding of microglial activation, which can only be achieved by using appropriate methodology.

Inflammation related PET studies in MS are traditionally focused on monitoring changes in glucose metabolism and the presence of activated microglia/macrophages in sclerotic lesions (Schiepers et al., 1997; Kiferle et al., 2011). [^18^F]FDG was recently used to evaluate the inflammation in the spinal cord in the EAE rat model (Buck et al., 2012). However, the basal uptake of glucose is elevated in the brain reducing the usability of [^18^F]FDG as a marker for brain lesions. Results of different stage patients indicate that [^18^F]FDG could be used to classify white matter lesions as either acute (hyper metabolism) or chronic (hypo metabolism) based on the glucose consumption (Paulesu et al., 1996; Dimitrakopoulou-Strauss et al., 2004; Buck et al., 2012). It is obvious that more specific markers are required to image the inflammation related metabolism in MS.

The majority of current PET tracers used to detect microglial activation utilize the expression of the peripheral benzodiazepine receptor (PBR), also known as the translocator protein TSPO (18 kDa) (Ryu et al., 2005; Ching et al., 2012). Translocator protein is expressed in the outer mitochondrial membrane. It was assumed to contribute through the cholesterol transportation into mitochondria regulating the rate of the synthesis of neurosteroids. However, these views have recently been challenged (Rupprecht et al., 2010; Selvaraj and Stocco, 2015). Gene-expression studies in the brain of rodents, primates, and humans have shown that TSPO expression is nearly absent in microglia patrolling the intact CNS parenchyma but rapidly increases in inflammation (Venneti et al., 2008; Ching et al., 2012). TSPO is highly expressed in activated microglia, in the choroid plexus and in reactive astrocytes, but its expression is globally low in the normal brain (Chauveau et al., 2008; Banati et al., 2014; Liu et al., 2014). These findings indicate that TSPO is a biomarker and an attractive target for the imaging microglial activation and reactive gliosis in cerebral inflammation (Rupprecht et al., 2010; Ching et al., 2012).

The isoquinoline carboxamide derivate PK11195 (N-butan-2-yl-1-(2-chlorophenyl)-N-methylisoquinoline-3-carboxamide), a nonbenzodiazepine ligand specifically binding to TSPO, has been widely used for its functional characterization and for the identification of its cellular origin in brain tissue (Banati et al., 1997, 2000; Chauveau et al., 2008). The issues regarding sensitivity and specificity of traditional PK11195 has been discussed (Venneti et al., 2008; Dickens et al., 2014; Boutin et al., 2015). Fortunately, recently developed radioligands such as DPA-714 (James et al., 2008; Chauveau et al., 2009), PBR28 (Imaizumi et al., 2007), PBR111 (Van Camp et al., 2010), SSR18075 (Chauveau et al., 2011), CLINME (Arlicot et al., 2008; Van Camp et al., 2010), and GE-180 (Dickens et al., 2014) have demonstrated better binding potency and bioavailability compared to the classical PK11195 and could overcome the problems of the classical tracers in MS and its models. A number of other TSPO targeting tracers have been developed to study the inflammation including but not limiting to DAA1106, FE-DAA1106, DPA-713, and vinpocetine, and reviewed by (Chauveau et al., 2008; James et al., 2008; Winkeler et al., 2010; Ciarmiello, 2011; Kiferle et al., 2011). Microglial activation was demonstrated in clinical MS studies with [^11^C]PK11195, unfortunately only in a limited number of patients (Banati et al., 2000; Debruyne et al., 2003; Versijpt et al., 2005; Vas et al., 2008). Radiotracer binding was increased in areas of acute and relapse-associated inflammation detected by classical Gd-DTPA enhanced T1-weighted MRI imaging (Rissanen et al., 2014). Interestingly, a significant increase in [^11^C]PK11195 binding was observed in activated microglia outside the histopathologically or MRI defined borders of MS plaques in both cerebral central gray-matter areas, which are not normally reported as sites of pathology in MS, as well as in normal appearing white matter (Banati et al., 2000; Debruyne et al., 2003). Unfortunately, 2nd generation TSPO targeting agents suffer from unexpected low binding status in over 30% of the population, which limits their use in clinics and demands genetic testing of the TSPO polymorphism. However, clinical studies have shown increased uptake with ^18^F-PBR111 and ^11^C-PBR28 in white matter lesions but not with all 2nd generation compounds like ^18^F-FEDAA1106. Additional studies are required to further investigate the specificity of these radiotracers for activated microglia over other activated glial cells. Overall, imaging of microglial activation in MS patients may serve as a complementary biomarker for disease progress (Abourbeh et al., 2012; Airas et al., 2015).

The type 2 cannabinoid receptor (CB2R) is part of the human endocannabinoid system and is involved in both central and peripheral inflammatory processes (Ehrhart et al., 2005; Pacher, 2006; Chiurchiù et al., 2014). CB2R can be found in immune cells, such as macrophages, perivascular T lymphocytes, astrocytes and reactive microglia, and it is thought to mediate anti-inflammatory as well as immunomodulatory effects (Docagne et al., 2008; Rodgers et al., 2013). 2-oxoquinoline and oxadiazolyle derivatives have been synthesized and radiolabeled with ^11^C and ^18^F, representing promising candidates for brain imaging in mice (Evens et al., 2009; Teodoro et al., 2013; Slavik et al., 2015). CB2R is almost undetectable in a healthy brain, whereas it is expressed in the activated glial cells (Stella, 2004; Cabral et al., 2008; Atwood and Mackie, 2010). This demonstrates that the effective PET ligands for CB receptors have the potential to act as biomarkers in the studies of pathophysiology of MS (Sanchez-Pernaute et al., 2008; Evens et al., 2009; Horti et al., 2010; Turkman et al., 2011; Vandeputte et al., 2011). In addition, new microglial targets, like P2X purinoceptor 7 (P2X7; Yiangou et al., 2006; Monif et al., 2009) and matrix metalloproteinases (Wagner et al., 2007; Iwama et al., 2011), have been explored for imaging of MS.

Overall, the benefits of PET contribute to the understanding of personalized status of MS patients, disease profiling, prognosis, and response, which are all combined in precision medicine. Specific biomarkers are the backbone for capturing the different aspects of MS heterogeneity, which could be useful for diagnosis, treatment stratification, and personalization of the therapeutic approach. Simplified, the precision medicine aims to provide the right drug with the right dose for the right indication in the right patient at the right time. Such as the case with the current 2nd generation TSPO markers, precision medicine relies on variability of genes, environment and life style of each person rather than on the data from large clinical trials. The customization of the treatment is based on the characterization of the genotype and phenotype induced effects on imaging in the individual patient. Biomedical imaging offers a great tool for mapping data from biomarkers, genomics, and physiology. There is a great interest for the monitoring of microglial activation in MS. However, the recent results with TSPO ligands suggest that the reactive astrocytes might increase the signal levels in MS (Lavisse et al., 2012). Since the role of reactive astrocytes in MS is recently of great interest, more specific markers are needed for reliable imaging of neuroinflammation (Rostami and Ciric, 2014; Zeis et al., 2015).

## Imaging astrocyte activation

Astrocytes are one of the most abundant cell types in the CNS. They have complex function ranging from supporting the surrounding neurons to the regulation of synaptic activity and BBB integrity (Sofroniew and Vinters, 2010). Although astrocytes are not immune cells *per-se* they can in specific conditions, such as in CNS inflammation, exert both pro- and anti-inflammatory effects on microglia (Min et al., 2006; Farina et al., 2007; Sofroniew, 2015).

Astrocytes were regarded to be non-participating bystanders in MS, responding secondarily to insults by undergoing astrogliosis and producing a glial scar (Brosnan and Raine, 2013). However, since the T-cell mediated immunity has been strongly associated with MS there are several plausible means by which astrocytes could contribute to autoimmunity. Astrocytes may facilitate immune cell extravasation into the CNS by releasing chemokines. They can modulate the activity of innate immune cells, such as microglia and inflammatory monocytes recruited to the CNS, by boosting their ability to promote neurodegeneration. Finally, astrocytes also have direct neurodegenerative functions mediated by the production of TNFα and nitric oxide (NO). However, these actions can also represent potential mechanisms by which astrocytes could reduce inflammation to promote remyelination (Claycomb et al., 2013).

Activation of glial cells is a common feature of MS as discussed earlier. Acetate is reported to accumulate into astrocytes and the [^11^C]acetate accumulation is increased in MS lesions (Takata et al., 2014). However, brain uptake of [^11^C]acetate is insufficient for obtaining a quantitative image of astrocytes' oxidative metabolism (Okada et al., 2013). To overcome this drawback benzyl [^11^C]acetate has been synthesized (Okada et al., 2013). Although the quantitative measurement remains under development, acetate is specific for astrocyte lipid metabolism (Brekke et al., 2015) and could serve as a marker for activated astrocyte metabolism in MS (Takata et al., 2014). In addition, ^18^F labeled derivative of acetate could increase the signal to noise ratio compared to ^11^C analog. It is expected that this tracer will be used in MS (Ponde et al., 2007).

One critical function of astrocytes is acting as sentinels and monitoring the BBB, a complex barrier composed of endothelial cells, astrocytes, pericytes, and myeloid cells such as perivascular macrophages and mast cells (Abbott et al., 2006). BBB functions as an anatomical mechanism for the highly selective passage of water, ions, nutrients, and cells from peripheral circulation into and out of the brain parenchyma (Abbott et al., 2006; Daneman and Rescigno, 2009; Larochelle et al., 2011). Under inflammatory conditions the BBB opens and it enables higher leukocyte passage into the CNS (Claycomb et al., 2013). Astrocytes play a critical role in shielding and protecting the CNS under inflammatory conditions (Voskuhl et al., 2009). Furthermore, astrocyte ablation has been shown to cause enhanced monocyte, but not T-cell, migration into the CNS (Toft-Hansen et al., 2011). To date, there is no clinically relevant PET tracer for BBB integrity, although several candidates have been proposed [^13^N]glutamate, [^82^Rb]Cl, or ^68^Gallium-ethylene-diamine-tetra-acetic acid (EDTA) (Saha et al., 1994; Wunder et al., 2012).

Imaging of astrocyte function is still a young field and it needs development of suitable imaging ligands. Astrocytes are involved in several neurological diseases and the main obstacle using imaging techniques has been the lack of proper tracers.

## Targets for PET imaging in MS

The recent increased availability of PET tracers to assess activated glial cells, disease pathology, and signaling pathways give PET a promising role in MS research. Since the underlying mechanisms of neurodegeneration and regeneration are still poorly understood the non-invasive techniques will enhance understanding these processes to develop better drug candidates, early diagnosis, and reliable monitoring of the treatment response. Several possible targets for PET imaging in MS are discussed in this section. These candidates may serve as more specific targets and may reveal some of the missing links in MS treatment and pathology, especially in the glial cell mediated actions.

Neuroinflammation is a dynamic and complex adaptive response process, which involves multiple cell types and various signaling routes, pathways, and receptors (Singhal et al., 2014). As discussed earlier, neuroinflammation can be imaged in MS. However, new tracers are needed to gain practical importance in clinics. The greatest potential may lay in the imaging of the dynamic interplay between neuroinflammation and the molecular mechanisms that contributes to the disease progression. The recent findings support three major neuroinflammation components in MS: astrogliosis, cytokine elevation, and significant changes in specific proteins, which offer a great variety of specific targets for imaging purposes.

TNF-α is associated with self-propagation of neuroinflammation and the expression of TNF-α is elevated in MS patients (Rossi et al., 2014). Microglia, inflammatory monocytes recruited to the CNS and astrocytes are major sources of TNF-α in CNS, interestingly proposing TNF-α expression as a marker in MS (Welser-Alves and Milner, 2013). PET tracers, like [^64^Cu]DOTA-etanercept and [^64^Cu]pegylated dimeric c(RGDyK), have been developed to target TNF-α in both acute and chronic inflammation in mice (Cao et al., 2007). TNF-α may be a target for MS imaging in the future. Overall, cytokines are highly related to oxidative stress in the brain (Di Penta et al., 2013). The expression of inducible nitric oxide synthase (iNOS) is increased in MS lesions, increasing generation of NO as well as reactive nitrogen species like peroxynitrite (Kröncke et al., 1998; Ortiz et al., 2013). The accumulation of these molecules induces lipid peroxidation, resulting in damage to DNA and neuronal degeneration (Haider et al., 2011).

In the healthy CNS tissues, the expression levels of iNOS are low but become highly expressed in astrocytes and neurons during inflammation (Saha and Pahan, 2006). In chronic pathology the reactive nitrogen species produced by iNOS are not efficiently eliminated, which leads to cellular dysfunctions (Fulda et al., 2010). The number of tracers for iNOS is minimal and the current [^18^F]NOS (6-(1/2)(2-[^18^F]fluoropropyl)-4-methylpyridin-2-amine) needs further modification and improvement. Importantly, the feasibility of iNOS PET imaging has been demonstrated in human inflammation (Herrero et al., 2012; Huang et al., 2015). In addition, active iNOS enzyme has been demonstrated in astrocytes in both acute and chronic active MS lesions and might therefore be an interesting target for imaging purposes (Liu et al., 2001).

The expression of another proinflammatory cytokine mediator, cyclooxygenase-2 (COX-2), is extensively increased in MS lesions and it has been tightly linked to increased iNOS expression (Rose et al., 2004). Furthermore, COX-2 expression was found in the cells expressing microglial marker, highlighting the importance of immune-derived cells. COX-2 has also been suggested to act as a link between neuroinflammation and glutamate mediated neuronal excitotoxicity (Kelley et al., 1999). These facts clearly indicate a need for methods to detect COX-2 expression. PET tracers for COX-2 have been developed, but the *in vivo* imaging properties have not been very effective (de Vries et al., 2003, 2008; Takashima-Hirano et al., 2011; Ji et al., 2013). The most promising COX-2 tracer so far is [^11^C]Rofecoxib (4-(4-methylsulfonylphenyl)-3-phenyl-5H-furan-2-one), demonstrating *in vitro* usability, but lacking necessary affinity for *in vivo* studies (Ji et al., 2013). Nevertheless, cyclooxygenases is presently an important target for PET tracer development.

Besides stimulating production of reactive oxygen species, cytokines are known to modulate the lipid metabolism and increase the production of neurodegeneration promoters such as eicosanoids and ceramides (Adibhatla and Hatcher, 2007). As previously mentioned, acetate is converted into fatty acid by acetyl-CoA synthase and [^11^C]acetate PET has proven useful for imaging in several diseases (Grassi et al., 2012). In addition, acetate is preferentially absorbed into astrocytes by the monocarboxylate transporter, which is overexpressed in MS (Nijland et al., 2014). Moreover, bioactive lipids exert significant effects on inflammation during autoimmunity targets or regulators of the immune response (Rinaldi et al., 2009). In addition, the appearance of cytosolic lipid synthesis is one the corner stones of macrophage foam cell formation (Matthäus et al., 2012). The intracellular concentrations of different individual lipids or the receptors involved the synthesis of particular bioactive lipids could reveal novel aspects of the disease progression (Mayo et al., 2014). Recently β-1,4-galactosyltransferase 6 (B4GALT6) was found to promote astrocyte activation and neuroinflammation during chronic EAE. The lactosylceramide (LacCer) synthesized by B4GALT6 in astrocytes controls the production of chemokines and cytokines, such as CCL2 and GM-CSF, which regulates the recruitment and activation of inflammatory monocytes and microglia and clearly highlights the importance of a specific lipid profile for disease progression (Mayo et al., 2014).

In summary, comprehensive profiling of lipid metabolism and the BBB function are likely to reveal new targets for therapeutic intervention in MS as well as for other neurological disorders where astrocyte activation contributes to disease pathology (Neu and Woelk, 1982; Pannu et al., 2005; Adibhatla and Hatcher, 2007; Wheeler et al., 2008; Kooij et al., 2012; Prüss et al., 2013). The imaging of specific bioactive lipids, receptors or enzymes that are involved in their synthesis may be novel targets for PET imaging.

## Biomarkers for the early phases of MS

In the search of better treatments for MS, cerebrospinal fluid (CSF) biomarkers have been used to identify high risk MS patients as well as patients with other neuronal disorders. Recently, high levels of astrocyte derived chitinase 3-like protein 1 (CHI3L1) were associated with the strong prediction of MS. This finding further demonstrates the increased importance of astrocyte activation and the specific role of astrocyte as a source for biomarkers in MS, already at the early disease phase.

The activated lipid metabolism in astrocytes demands increased acetate and lipid transportation (Lev, 2012). ATP and glutamate stimulation can significantly enhance the dynamin-independent endocytosis and their receptors control the microglial physiology and pathology (Jiang and Chen, 2009). For example ATP related purinergic receptors control microglial cytokine release among several other functions (Sperlágh and Illes, 2007). Moreover, purinergic pathways regulate neuroinflammation (Burnstock, 2008). The increasing evidence suggests that the P2X7 receptor is an interesting neuroinflammation associated molecular target (Lister et al., 2007; Monif et al., 2009; Gandelman et al., 2010). PET tracers have been developed to image P2X7 receptor, [^11^C]A-740003 (N-[1-[[(Cyanoamino)(5-quinolinylamino)methylene]amino]-2,2-dimethylpropyl]-3,4-dimethoxybenzeneacetamide) and [^11^C]GSK1482160 ((S)-N-(2-chloro-3-(trifluoromethyl)benzyl)-1-[^11^C]methyl-5-oxopyrro-lidine-2-carboxamide; Janssen et al., 2014; Gao et al., 2015). Purinergic system might serve as a sensitive target for MS imaging.

Furthermore, the adenosine receptors, whose expression is modulated by microglial activation, moderate immune function (Haskó et al., 2008; Orr et al., 2009; Domercq et al., 2013; Luongo et al., 2014). Especially A_2A_ receptors are up-regulated during inflammation (Rissanen et al., 2013). It is clear that adenosine signaling play a significant role in MS as a neuromodulator and the clinical studies with [^11^C]TMSX (7-methyl-[^11^C]-(E)-8-(3,4,5-Trimethoxystyryl)-1,3,7-trimethylxanthine) PET will likely open new perspective to develop new tracers to this target in the future (Rissanen et al., 2015).

In addition, the cholinergic system shows decreased function in MS patients (Kooi et al., 2011). PET imaging studies of cholinergic activity may define which patient will respond to the treatment which will further increase the knowledge of MS. A similar approach has been already used in Alzheimer's disease using radiolabeled choline derivatives and these techniques could be easily transferred to MS clinical research (Volkow et al., 2001; Rinne, 2003; Kooi et al., 2011).

The cannabinoid receptors CB2 are expressed in very low levels in a healthy brain, but the expression increases during microglial activation (Benito et al., 2007). CB2 is an interesting target for PET imaging in MS models especially with [^11^C]A836339 (2,2,3,3-Tetramethylcyclopropanecarboxylic acid [3-(2-[11C]methoxyethyl)-4,5-dimethyl-3H-thiazol-(2Z)-ylidene]amide) and [^11^C]NE40. Recently there has been great progress in developing new tracers for this target (Docagne et al., 2008; Horti et al., 2010; Evens et al., 2011, 2012; Slavik et al., 2015; Yrjölä et al., 2015).

**Figure 5:**
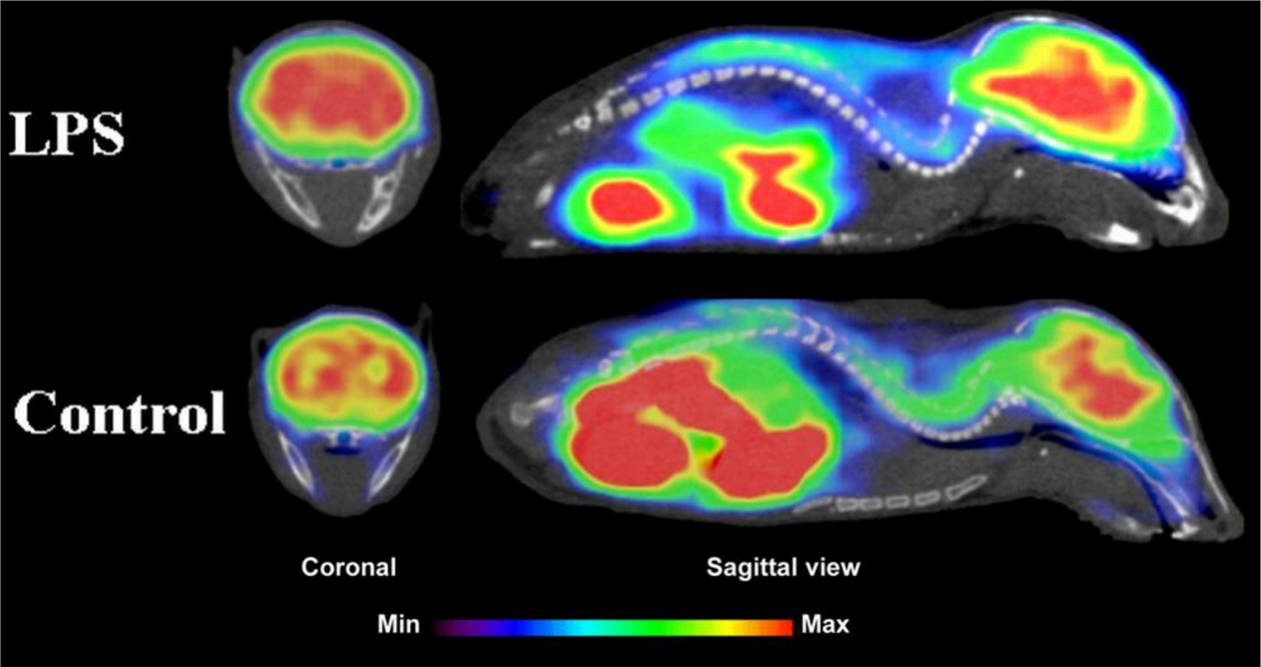
**Coronal and sagittal sections of fused PET and CT images in 10 days old pups of mice**. PET studies using [^18^F]FPEB show enhanced mGluR5 expression in the brain of the pups, whose mothers were injected with LPS compared to saline injection (control). Coronal slices show highest accumulation in the hippocampal area of the mouse, whose mother had LPS administration. Sagittal images show spine based on CT images and high accumulation of [^18^F]FPEB in the brain and gut. Modified from Arsenault et al. (2014).

During inflammation microglia will release glutamate in response to the production of reactive oxygen species (ROS; Bal-Price and Brown, 2001; Brown and Neher, 2010; Takaki et al., 2012). The cysteine-glutamate exchange modulates the release of ROS and cytokines which impairs the function of glutamate transporters and leads to increased extracellular glutamate levels as well as excitotoxicity (Rao et al., 2003; Matute et al., 2006). Metabotropic glutamate receptors (mGluRs) are transmembrane proteins that are expressed in glial cells and play a pivotal role in cell function and glial-neuronal co-operation (Kritis et al., 2015). Immunohistochemical analyses have revealed that mGluR5 is expressed in reactive astrocytes surrounding the MS lesion site and the expression is higher than in non-activated astrocytes (Geurts et al., 2003). In addition, the activation of mGluR5 reduced the microglial activation in an inflammation model (Byrnes et al., 2009; Loane et al., 2009). During the last 15 years the subtype selective allosteric modulators have been identified for different mGluRs (see Figure 5; Zhang and Brownell, 2012). Many PET tracers have been synthesized by radiolabeling the derivatives of MPEP and MTEP and to date over 15 mGluR5 targeting PET ligands have been reported (Mu et al., 2010; Zhang and Brownell, 2012). Tracers like [^18^F]FPEB ((3-[^18^F]Fluoro-5-(2-pyridinylethynyl)benzonitrile) have been already developed for automated synthesis and evaluated in humans (Lim et al., 2014).

Monoamine oxidase type B located in the outer membrane of mitochondria and is expressed in astrocytes, where its activity is increased in neurodegenerative diseases (Mallajosyula et al., 2008; Veitinger et al., 2014). It catalyzes the deamination reaction thus modulating neurotransmitter concentrations and has been a major target for drug development, especially in movement related diseases (Talati et al., 2009; Deftereos et al., 2012). The ^11^C-L-deprenyl indicates increased monoamine oxidase type B content in reactive and proliferating astrocytes in AD (Gulyás et al., 2011). Results from the MS patients studies are likely to be published soon (Hurley, 2015).

The above exploration shows that the combined PET imaging of activated microglia and astrocytes is presently of special interest in MS research.

## PET as a tool for precision medicine in MS

More specific features of MS lesions have been described in parallel with the identification of body fluid markers such as CHI3L1 and B4GALT6. Recent progress with biomarkers and imaging tracers suggests that precision medicine is becoming a reality in MS. The prevalence of MS is increasing and there is relatively little data available to personalize the treatments and increase the cost effectiveness. Sophisticated tools are needed to handle the complex data to obtain more detailed insight of the clinical status of the patient's condition. The combined information from various biomarkers and imaging studies can be used to predict the disease evolution individually. PET imaging can provide precise data for the cross-roads of multiple fields, like biomedical imaging, pharmacology, neurology, genomics etc. Achieving precision medicine in MS requires high quality data, large samples, and consistent interdisciplinary approach.

## Conclusion

Inflammation and glial activation play an important role in numerous neurodegenerative diseases, such as Alzheimer's disease, Parkinson disease, amyotrophic lateral sclerosis and MS. Although factors inducing inflammation vary between diseases, there is evidence of greatly converging mechanisms for the sensing, transduction, and amplification of inflammatory processes that eventually lead to the production of neurotoxic mediators. PET imaging provides a powerful method for dynamic imaging of these events. The full potential of PET is not yet recognized, mainly due to the lack of validated tracers; the complicated and costly process of validating new tracers needs partnerships, human resources, expertise, funding, and access to patients, but it is something that needs to be focused on to obtain the essential information of the biological processes in disease pathology. This will ultimately produce more reliable diagnosis, better treatments and effective prevention methods for MS. The role of PET imaging will increase in clinics, when onsite cyclotrons, the development of new tracers, and imaging equipment become available.

[^18^F]-FDG is still the most extensively used PET imaging tracer for inflammation even though it tends to produce controversial results. New imaging tracers for TSPO ([^11^C]PK11195, [^11^C]PBR28, etc.) have gained a great interest for detection of inflammation and evaluation of therapy. Using these new tracers, PET imaging has greatly improved our understanding of the mechanism of inflammation and increased the diagnostic specificity and accuracy of inflammation. As summarized in Table 2, various radiopharmaceutical approaches have been developed for PET imaging to detect inflammation, including biomarkers targeting to specific receptors and lipid metabolism.

**Table 2:** **Examples of PET tracers in MS research**.

**MS characteristic**	**Target**	**Compound**	**Structure**	**Stage in MS imaging**	**References**
Axonal degeneration	GABA_A_ receptor	^18^F-flumazenil (K_i_ ~6.0 nM)	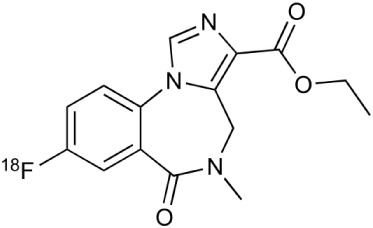	Clinical studies in MS patients are ongoing.	Banati et al., 1997, 2000; Ohyama et al., 1999; Barkhof et al., 2009; Pascual et al., 2012
		^11^C-MP4A	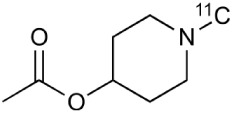	Clinical studies in AD patients.	Virta et al., 2011; Garibotto et al., 2013; Lund et al., 2013
		^11^C-Ro15-4513	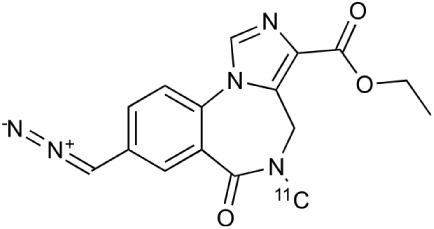	Subtype specific ligand. No studies with MS patients.	Halldin et al., 1992; Quelch et al., 2015
	Glucose metabolism	^18^F-FDG	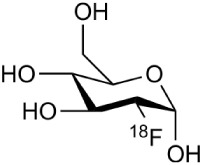	Studies with MS patients conducted.	Kuhlmann, 2002; Buck et al., 2012; Maffione et al., 2014; Rudroff et al., 2014
Demyelination and remyelinination	Choline metabolism	^11^C-Choline	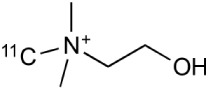	Clinically approved for cancer imaging.	Stankoff et al., 2006, 2011
		^11^C-BMB	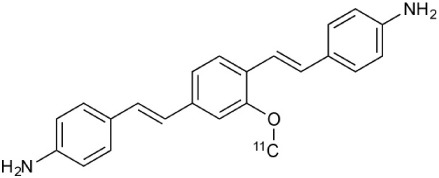	Studies with MS patients conducted.	Stankoff et al., 2006, 2011
		^11^C-CIC	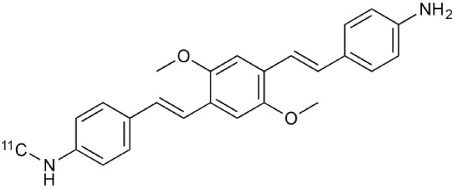	Studies with preclinical MS rodent models.	Wang et al., 2009; de Paula Faria et al., 2014a,b; Ellwardt and Zipp, 2014
		^11^C-PIB (K_i_ ~1.9 nM)	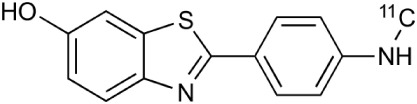	Studies with MS patients.	de Paula Faria et al., 2014a,b
		^11^C-MeDAS	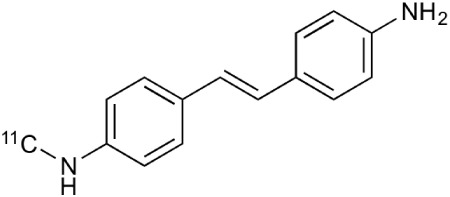	Studies with MS mouse models.	Wu et al., 2010, 2013; de Paula Faria et al., 2014a,b
Glial activation	TSPO	^11^C-PK11195 (K_i_ ~9.3 nM)	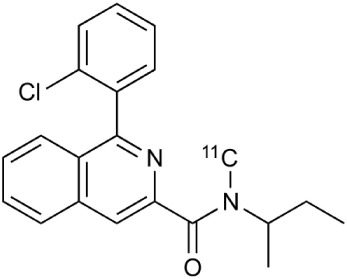	Studies with MS patients and early stage MS m patients conducted.	Debruyne et al., 2003; Versijpt et al., 2005; Politis et al., 2012; Rissanen et al., 2014; Giannetti et al., 2015
		^11^C-DAA1106 (K_i_ ~0.28 nM)	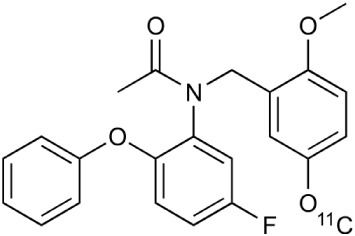	Preclinical models. Clinical studies have been conducted with healthy volunteers.	Maeda et al., 2004; Venneti et al., 2008; Brody et al., 2014
		^18^F-FE-DAA1106 (K_i_ ~0.08 nM)	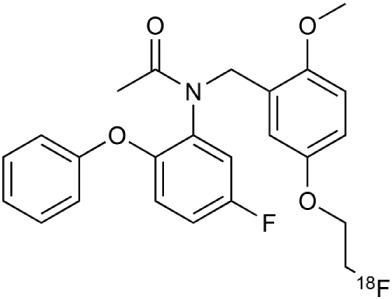	Clinical studies in MS patients conducted.	Ji et al., 2008; Takano et al., 2013
		^11^C-DPA-713 (K_i_ ~4.7 nM)	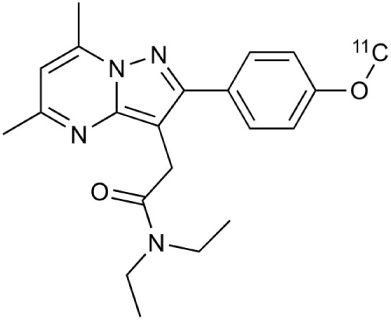	Preclinical models in MS. Clinical studies with healthy patients and patients with inflammation.	Boutin et al., 2007a; Endres et al., 2009; Coughlin et al., 2014
		^18^F-DPA-714 (K_i_ ~7.0 nM)	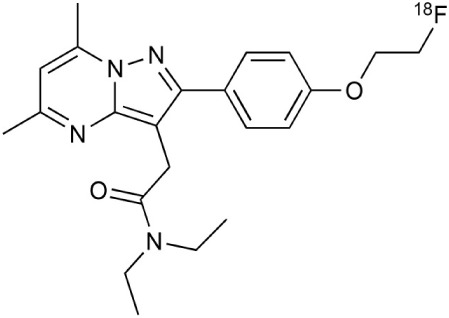	Preclinical models in MS. Clinical studies in AD patients.	Peyronneau et al., 2013; Golla et al., 2015
		^18^F-PBR28 (K_i_ ~4.6 nM)	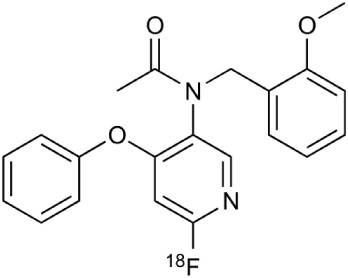	Clinical studies in MS patients.	Oh et al., 2011; Moon et al., 2014; Park et al., 2015
		^18^F-PBR111 (K_i_ ~4.5 nM)	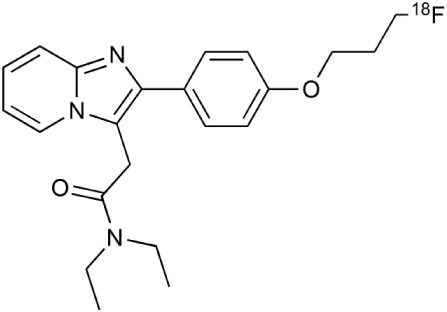	Clinical studies in MS patients.	Mattner et al., 2013; Colasanti et al., 2014
		^11^C-CLINME (K_i_ ~8.5 nM)	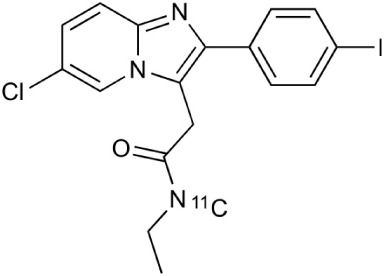	Preclinical in MS. Clinical studies with acuteneuroinflammation.	Boutin et al., 2007b; Van Camp et al., 2010
		^11^C-vinpocetine	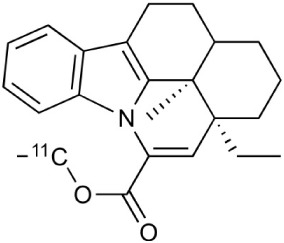	Clinical studies in MS patients.	Vas et al., 2008; Oh et al., 2011
		^18^F-GE180 (K_i_ ~0.87 nM)	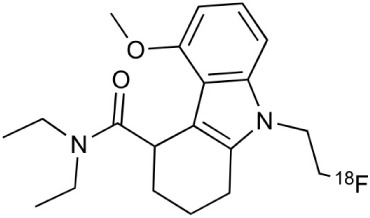	Preclinical studies in MS models. Clinical studies with inflammation.	Wadsworth et al., 2012; Dickens et al., 2014; Airas et al., 2015
	CB2r	^8^F-GW405833	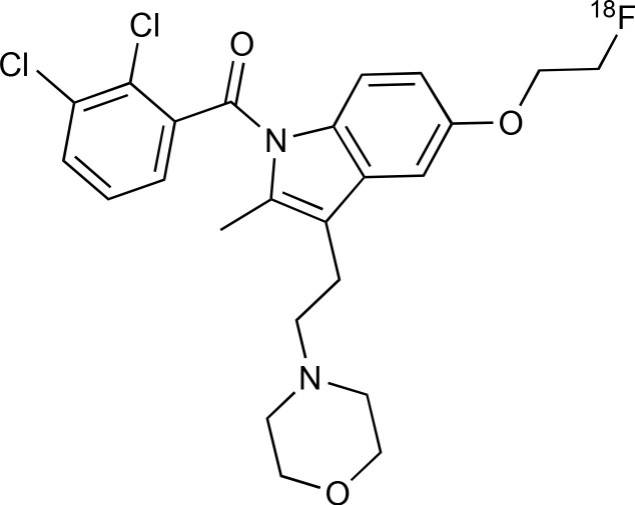	Preclinical models.	Vandeputte et al., 2011
		^11^C-A-836339	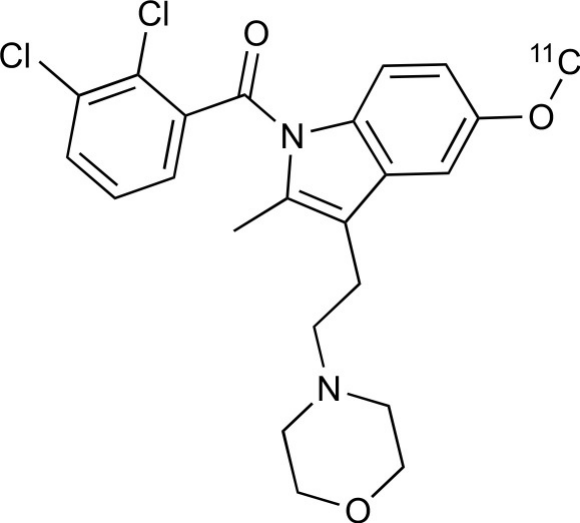	Preclinical models.	Horti et al., 2010
		^11^C-KD2	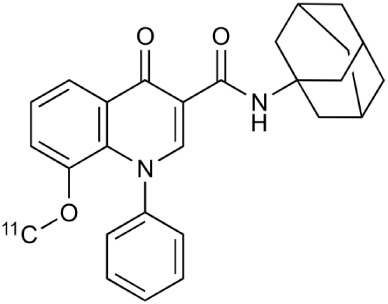	Preclinical models.	Mu et al., 2013
	P2X7 receptor	^11^C-A-740003	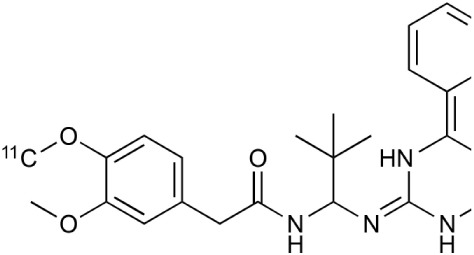	Rodent baseline.	Janssen et al., 2014
	Matrix metalloproteinases	^18^F-CGS27023A	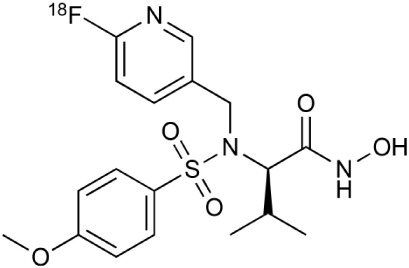	Preclinical models.	Wagner et al., 2007, 2009
		^18^F-CGS25966	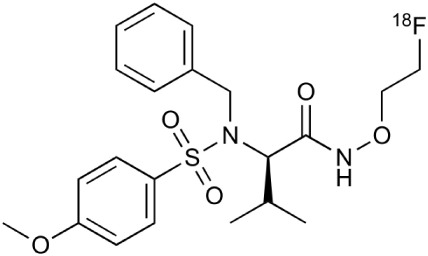	Preclinical models.	Wagner et al., 2007
	Monoamine oxidase type B	^11^C-l-deprenyl	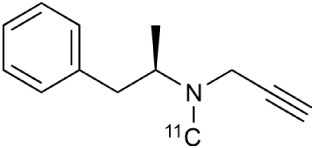	Peclinical models. Clinical studies in ALS patients.	Johansson et al., 2007; Gulyás et al., 2011
	Lipid metabolism	^18^F-Acetate	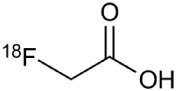	Preclinical models.	Marik et al., 2009
		^11^C-Acetate	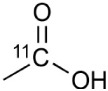	Clinical studies in MS patients.	Takata et al., 2014
	Metabotropic glutamate receptor subtype 5	^11^C-ABP688	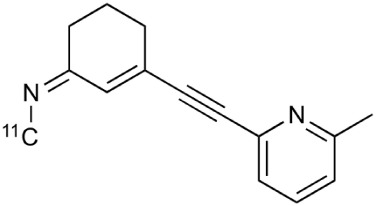	Clinical studies. No MS studies.	Ametamey et al., 2006; DeLorenzo et al., 2015
		^11^C-MPEP	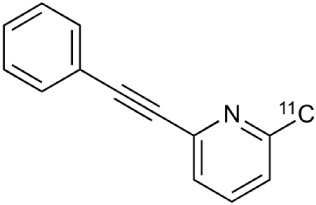	No MS imaging published.	Yu et al., 2005
		^18^F-FBEP	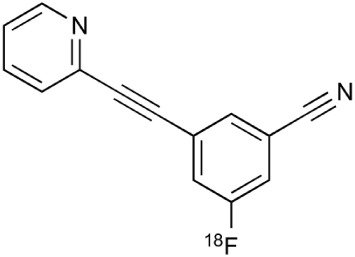	Clinically validated. No MS imaging published.	Wang et al., 2007; Wong et al., 2013
	Induced nitric oxide synthetase	^18^F-NOS	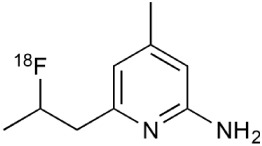	No MS imaging published. Clinical studies with inflammation.	Herrero et al., 2012
	Cyclooxygenase-2	^11^C-Rofecoxib	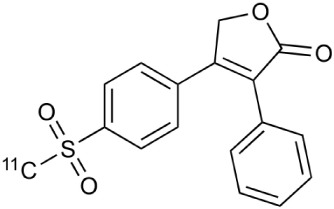	Preclinical evaluation with inflammation model.	de Vries et al., 2008

So far, the clinical PET studies in MS are limited to evaluation of two biological processes: glucose metabolism and inflammation. It is clear that the use of combined PET/MR imaging is increasing also in MS research. One of the main interests is to develop combined imaging markers and methods for MS pathology to stage, cell type and record activity related changes in lesions. However, presently PET imaging is relatively expensive and it also requires sophisticated quantification, which demands special software and skilled operators. MS is a complex disease, which remains difficult to treat before more specific disease mechanisms are revealed. PET research community is looking for the first ligands to be recommended for routine clinical practice in MS diagnosis and follow up of therapy. PET has already shown to be one of the most sophisticated, sensitive, reliable, effective, and safest tools for the monitoring of several cancers in clinics and there is no reason why it could not be same in the neurodegenerative disorders as well. Clinical imaging and research modalities should be combined to expand the knowledge of clinical findings, genetics, phenotyping, pharmacology, and drug targeting. Advanced imaging technologies, including PET, could be used to reveal the causes of MS rather than concentrating on correlations. MS is a complex and heterogeneous disease, which could benefit from precision medicine in the future. The genomic approach can be used to individualize the imaging data as presently done with 2nd generation TSPO markers ([^11^C]PBR28 etc.). Astrocyte activation and their ability to modulate the complex neuronal network and inflammation related pathways have a great potential to reveal disease stage specific markers for personalized medicine. Despite the astrocyte related research in MS is still in early stages, and the recent promising results suggest new techniques to diagnose, monitor and treat this cruel disease. The combined pathogenic characteristics of MS are still unknown and the key to prevent and cure this devastating disease is still waiting for discovery.

## Author contributions

PP did literature search, prepared tables and participated writing. MJ participated literature search, writing and composing the manuscript. FQ did critical evaluation of the manuscript. AB participated writing, preparation of figures, and final evaluation of the manuscript.

### Conflict of interest statement

The authors declare that the research was conducted in the absence of any commercial or financial relationships that could be construed as a potential conflict of interest.
